# Smoking cessation, alcohol intake and transient increase in the risk of metabolic syndrome among Japanese smokers at one health checkup institution

**DOI:** 10.1186/1471-2458-9-263

**Published:** 2009-07-27

**Authors:** Asahi Hishida, Atsushi Koyama, Akiko Tomota, Shirou Katase, Yatami Asai, Nobuyuki Hamajima

**Affiliations:** 1 Department of Preventive Medicine/Biostatistics and Medical Decision Making, Nagoya University Graduate School of Medicine, Nagoya, Japan; 2 Institution for Disease Prevention and Health Checkup, Seirei Mikatabara Hospital, Hamamatsu, Japan

## Abstract

**Background:**

Metabolic syndrome (MetS) is potentially effective measures to identify individuals at risk of coronary heart disease (CHD) and type 2 diabetes. To verify the hypothesis that smoking cessation may increase the risk of MetS, a follow-up study taking drinking habit into account was conducted for the examinees at one health checkup institution.

**Methods:**

Subjects were the examinees who visited the Institution for Disease Prevention and Health Checkup, Seirei Mikatabara Hospital for annual health checkup from January 2003 to December 2006. Among them, 5,872 smokers (5,479 men, 93.3%) free from MetS at the first year in two consecutive years were selected. For the long term follow-up, the risk of MetS among those who maintained their nonsmoking status for 1 or 2 additional years was evaluated.

**Results:**

Relative to non-quitters, quitters showed a significantly elevated adjusted hazard ratio (aHR) of MetS in two consecutive years (aHR = 2.09, 95% confidence interval: 1.43–3.04, *P *< 0.001). The aHR was higher among the quitters who had a drinking habit at the first year (aHR = 2.42, 95% CI: 1.48–3.94, *P *< 0.001). Analyses for 1 or 2 additional years of follow-up revealed that this significant increase in risk of MetS was transient.

**Conclusion:**

The present study revealed that smoking cessation elevated the risk of MetS significantly, especially among drinkers. Although this detrimental effect of smoking cessation was found to be during only a short term, our results suggested that we should take measures, presumably including interventions for alcohol cessation, not to expose smoking quitters to this adverse effect. Further investigations are required to confirm our findings.

## Background

Facing the era of aging society, Japanese nations have come to pay more and more attention to the metabolic syndrome (MetS), which was recently defined by National Cholesterol Education Program (NECP) to help identify individuals at risk of coronary heart disease (CHD) and type 2 diabetes [1]. In the preceding decades, lifestyles changed: sedentary lifestyles became more and more common; people got used to ride a car when they moved, where they had less opportunity to walk, contributing to the increasing development of diabetes or MetS [2].

Recently, it was reported by Japanese researchers that cigarette smoking increased the risk of MetS, and they also found that body weight gain was higher in smokers who quit smoking compared with never smokers [3]. It is well known that people gain weight after quitting smoking; from many clinical experiences, it seemed probable that smoking cessation would raise the risk of MetS.

Some studies reported a possible association between alcohol intake and risk of MetS [4], but this association is still controversial. Other reports also demonstrated the beneficial effect of alcohol intake on the risk of MetS, suggesting that this relationship is rather complex [5,6]. Alcohol consumption was reported to have favorable effects on plasma high density lipoprotein cholesterol (HDL-C) levels [7] and insulin sensitivity, as well as unfavorable effects to increase plasma triglyceride (TG) levels or elevate blood pressure (BP). The association between alcohol and weight gain is still discussed.

In this study, to verify the hypothesis that smoking cessation may increase the risk of MetS and alcohol intake might also modulate its risk, a follow-up study was conducted for health checkup examinees at Institution for Disease Prevention and Health Checkup, Seirei Mikatabara Hospital.

## Methods

### Study Subjects

The study subjects were the health checkup examinees who visited the Institution for Disease Prevention and Health Checkup, Seirei Mikatabara Hospital for annual health checkup from January 2003 to December 2006. In total, 15,508 examinees (10,243 men, 66.0%) in year 2003, 15,584 (10,123 men, 65.0%) in 2004, 16,322 (10,492 men, 64.3%) in 2005, and 16,586 (10,630 men, 64.1%) in 2006 visited this annual health checkup. Most of the examinees visited the institute for health checkup annually. Among them, 5,872 subjects (5,479 men, 93.3%) who visited it in two consecutive years and those who smoked and did not suffer from MetS or diabetes mellitus (DM) in the first year were selected from the whole examinees. Among these selected examinees, 185 subjects (177 men, 95.7%) became MetS. Table 1 shows the characteristics of the selected study subjects. For the long term follow-up, the risk of MetS among those who smoked in the first year and quitted smoking in the second year, and maintained their nonsmoking status for one or two additional years was evaluated.

**Table 1:** Baseline characteristics of the selected study subjects according to smoking status.

	**Non-quitter**			**Quitter**		
	**Men**	**Women**	**Total**	**Men**	**Women**	**Total**
***n***	4967	347	5314	512	46	558
**Age^a^**	50.2 ± 8.6	49.4 ± 8.1	50.1 ± 8.6	51.5 ± 9.2	50.1 ± 8.3	51.4 ± 9.1
						
***Lifestyle behaviors***						
***Habitual drinkers***	3765 (75.8%)	174 (50.1%)	3939 (74.1%)	379 (74.0%)	16 (34.8%)	395 (70.8%)
***Current smokers***	4967 (100.0%)	347 (100.0%)	5314 (100.0%)	512 (100.0%)	46 (100.0%)	558 (100.0%)
						
***Components for MetS***						
**HDL cholesterol (mg/dL)**						
**< 40**	554 (11.2%)	10 (2.9%)	564 (10.6%)	53 (10.4%)	3 (6.5%)	56 (10.0%)
**≥ 40**	4413 (88.8%)	337 (97.1%)	4750 (89.4%)	459 (89.6%)	43 (93.5%)	502 (90.0%)
						
**Fasting triglyceride (mg/dL)**						
**< 150**	3612 (72.7%)	300 (86.5%)	3912 (73.6%)	391 (76.4%)	39 (84.8%)	430 (77.1%)
**≥ 150**	1355 (27.3%)	47 (13.5%)	1402 (26.4%)	121 (23.6%)	7 (15.2%)	128 (22.9%)
						
**Fasting serum glucose (mg/dL)**						
**< 110**	4555 (91.7%)	327 (94.2%)	4882 (91.9%)	471 (92.0%)	45 (97.8%)	516 (92.5%)
**≥ 110**	412 (8.3%)	20 (5.8%)	432 (8.1%)	41 (8.0%)	1 (2.2%)	42 (7.5%)
						
**Systolic blood pressure (mmHg)**						
**< 130**	3957 (79.7%)	308 (88.8%)	4265 (80.3%)	408 (79.7%)	40 (87.0%)	448 (80.3%)
**≥ 130**	1010 (20.3%)	39 (11.2%)	1049 (19.7%)	104 (20.3%)	6 (13.0%)	110 (19.7%)
						
**Diastolic blood pressure (mmHg)**						
**< 85**	4340 (87.4%)	324 (93.4%)	4664 (87.8%)	449 (87.7%)	42 (91.3%)	491 (88.0%)
**≥ 85**	627 (12.6%)	23 (6.6%)	650 (12.2%)	63 (12.3%)	4 (8.7%)	67 (12.0%)
						
**Body mass index**						
**< 22**	2013 (40.5%)	198 (57.1%)	2211 (41.6%)	209 (40.8%)	25 (54.3%)	234 (41.9%)
**22 ≤ < 25**	2113 (42.5%)	104 (30.0%)	2217 (41.7%)	223 (43.6%)	14 (30.4%)	237 (42.5%)
**≥ 25**	841 (16.9%)	45 (13.0%)	886 (16.7%)	80 (15.6%)	7 (15.2%)	87 (15.6%)

^a^Data is shown as mean ± standard deviation (SD).

### Data collection

Annual health checkup at the institute included medical examination and interviews about medical history and health-related behavior, consisting of data on smoking status, alcohol intake, and regular physical activity. BP measurements and anthropometric measurements of height and weight were done by trained examiners. Fasting serum glucose (FSG), HDL-C, and TG levels were determined in serum samples obtained at the time of the medical examination.

### Diagnostic criteria

The diagnosis of MetS was defined according to the modified Japanese criteria (criteria defined by Japanese Society for the Study of Obesity (JASSO)) [8] as follows: For the major criteria, overall obesity was defined as body mass index (BMI) of ≥ 25 kg/m^2 ^instead of waist circumference (WC) as described in the previous Japanese report [3], because we could not obtain the data of WC in this population. For the minor criteria, we investigated the presence of three metabolic risk factors: hypertension, dyslipidemia, and impaired fasting glucose. Hypertension represented elevated BP: systolic BP (sBP) ≥ 130 mmHg and/or diastolic BP (dBP) ≥ 85 mmHg. Dyslipidemia represented hypertriglyceridemia (fasting TG ≥ 150 mg/dl), and/or low HDL-C (< 40 mg/dl). Impaired fasting glucose represented hyperglycemia: FSG concentration ≥ 110 mg/dl. Those who met the obesity criteria (major criteria) and no less than two of three metabolic risk factors (minor criteria: hypertension, dyslipidemia and impaired fasting glucose) were diagnosed as having MetS. Those with FSG concentration ≥ 126 mg/dl at baseline were diagnosed as having DM and excluded. Those who became diabetic in the second year were excluded from MetS. Smoking cessation was defined for those who stated to be current smokers at questionnaire in the first year, and stated to have quitted smoking or to be no current smokers in the second year. Those who smoked in the first year and quitted in the second year are defined as smoking "quitters," and the rest are called "non-quitters." Among the quitters, those who stated to be habitual alcohol drinkers in the second year were defined as "drinkers."

### Evaluation of MetS components

We categorized the examinees who became MetS in the second year among smoking quitters (n = 33) according to the combinations of fulfilled MetS criteria components in the first year (before cessation) and in the second year (after cessation), and evaluated which component contributed to the occurrence of MetS in each case.

### Statistical analysis

The adjusted hazard ratio (aHR) and 95% confidence interval (95% CI) were estimated with a Cox's proportional hazard model adjusted by age and sex. Differences of the MetS parameters of two groups were tested by a multiple linear regression model. Statistical analysis was conducted with STATA version 7 (STATA Corporation, College Station, TX).

### Ethics

This study was approved by the Ethics Committee of Seirei Mikatabara Hospital.

## Results

Table 2 shows the risk of MetS according to smoking status. When those who continued smoking were defined as the reference, those who quitted smoking showed significantly or nearly significantly elevated aHR for MetS in any two consecutive years (aHR = 2.09, 95% CI: 1.43–3.05, *P *< 0.001 in years 2003–2006 total: aHR = 2.60, 95% CI: 1.34–5.04, *P *= 0.005 in years 2003–2004; aHR = 1.84, 95% CI: 1.01–3.37, *P *= 0.048 in years 2004–2005; aHR = 1.92, 95% CI: 0.94–3.93, *P *= 0.073 in years 2005–2006). To investigate the main factor contributing to this risk elevation of MetS among smoking quitters, we next compared each MetS parameters of smoking quitters with those of non-quitters by multiple linear regression model (Table 3, 4). In total, increase in HDL-C levels were significantly higher in smoking quitters than in non-quitters (*P *< 0.001), while increases in FSG (*P *< 0.001), BP (both sBP and dBP) (*P *< 0.001 and *P *< 0.001, respectively) and BMI (*P *< 0.001) were significantly higher in smoking quitters than in non-quitters (Table 3). To exclude the possibility that changes in these parameters were just the reflection of BMI increase, we calculated the differences of these parameters adjusted by sex, age and increase in BMI by multiple regression model, all of which still remained significant; HDL-C levels (*P *< 0.001); FSG (*P *< 0.001); sBP (*P *< 0.001) and dBP (*P *< 0.001) (Table 4).

**Table 2:** Adjusted hazard ratio (aHR)^a ^and 95% confidence intervals (95% CIs) of metabolic syndrome according to smoking status.

	**Men**		**Women**		**Total**	
	
	**Non-quitter**	**Quitter**	**Non-quitter**	**Quitter**	**Non-quitter**	**Quitter**
***n***	4972	496	342	62	5314	558
						
**MetS**	145	32	7	1	152	33
**aHR**	1	2.20	1	0.75	1	2.09
**95% CI**	Reference	1.50–3.23***	Reference	0.09–6.14	Reference	1.43–3.05***
						
**Major Criteria (BMI)**	881	116	57	7	938	123
**aHR**	1	1.36	1	0.68	1	1.29
**95% CI**	Reference	1.12–1.66**	Reference	0.31–1.48	Reference	1.07–1.56**
						
**Hypertension**	1150	165	43	6	1193	171
**aHR**	1	1.34	1	0.75	1	1.30
**95% CI**	Reference	1.14–1.58***	Reference	0.32–1.76	Reference	1.11–1.53**
						
**Dyslipidemia**	1647	170	43	4	1690	174
**aHR**	1	1.04	1	0.50	1	1.02
**95% CI**	Reference	0.89–1.22	Reference	0.18–1.40	Reference	0.87–1.19
						
**Impaired Fasting Glucose**	504	68	17	7	521	75
**aHR**	1	1.28	1	2.22	1	1.32
**95% CI**	Reference	0.99–1.65	Reference	0.92–5.37	Reference	1.04–1.69*
						
**Minor Criteria^b^**	584	84	15	3	599	87
**aHR**	1	1.37	1	1.05	1	1.35
**95% CI**	Reference	1.09–1.72**	Reference	0.30–3.64	Reference	1.08–1.69**

*: *P *< 0.05, **: *P *< 0.01, ***:*P *< 0.001.

^a^Adjusted for sex and age (total), or age (men and women).

^b ^Minor criteria include hypertension, dyslipidemia and impaired fasting glucose.

**Table 3:** Multiple linear regression coefficients of smoking cessation for changes in metabolic syndrome parameters after smoking cessation.

	**Men**		**Women**		**Total**	
	**Coefficient**	**95% CI**	**Coefficient**	**95% CI**	**Coefficient**	**95% CI**
***All Subjects***						
(quitter 558, non-quitter 5314)						
						
**HDL cholesterol (mg/dL)**	2.3	(1.6, 3.0)***	1.6	(-1.1, 4.3)	2.2	(1.6, 2.9)***
**Fasting triglyceride (mg/dL)**	3.6	(-3.6, 10.8)	7.5	(-5.9, 20.9)	3.9	(-2.9, 10.7)
**Fasting serum glucose (mg/dL)**	2.3	(1.7, 3.0)***	2.7	(0.6, 4.8)*	2.4	(1.8, 3.0)***
**Systolic blood pressure (mmHg)**	4.0	(2.8, 5.1)***	0.0	(-3.6, 3.6)	3.7	(2.6, 4.7)***
**Diastolic blood pressure (mmHg)**	2.7	(1.8, 3.5)***	0.6	(-2.2, 3.5)	2.5	(1.7, 3.3)***
**Body mass index**	0.5	(0.5, 0.6)***	0.3	(0.1, 0.5)	0.5	(0.4, 0.6)***
						
***MetS***						
(quitter 33, non-quitter 152)						
						
**HDL cholesterol (mg/dL)**	3.7	(1.2, 6.1)**	13.3	(-32.2, 58.7)	3.9	(1.5, 6.3)**
**Fasting triglyceride (mg/dL)**	-10.9	(-46.7, 25.0)	-36.9	(-190.7, 116.9)	-11.9	(-46.9, 23.0)
**Fasting serum glucose (mg/dL)**	3.0	(0.1, 5.8)*	17.5	(-53.2, 88.1)	3.3	(0.5, 6.2)*
**Systolic blood pressure (mmHg)**	2.9	(-2.4, 8.3)	11.1	(-20.4, 42.5)	3.0	(-2.2, 8.2)
**Diastolic blood pressure (mmHg)**	2.2	(-1.8, 6.1)	24.3	(-18.2, 66.8)	2.7	(-1.2, 6.6)
**Body mass index**	0.6	(0.3, 0.9)***	3.1	(1.0, 5.1)*	0.6	(0.4, 0.9)***
						
***without MetS***						
(quitter 525, non-quitter 5162)						
						
**HDL cholesterol (mg/dL)**	2.2	(1.5, 2.9)***	1.4	(-1.3, 4.1)	2.2	(1.5, 2.9)***
**Fasting triglyceride (mg/dL)**	3.9	(-3.5, 11.3)	8.4	(-5.2, 22.0)	4.2	(-2.7, 11.2)
**Fasting serum glucose (mg/dL)**	2.2	(1.5, 2.8)***	2.4	(0.3, 4.5)*	2.2	(1.5, 2.8)***
**Systolic blood pressure (mmHg)**	3.9	(2.7, 5.0)***	-0.1	(-3.7, 3.5)	3.5	(2.4, 4.6)***
**Diastolic blood pressure (mmHg)**	2.6	(1.7, 3.5)***	0.2	(-2.7, 3.1)	2.4	(1.6, 3.2)***
**Body mass index**	0.5	(0.4, 0.6)***	0.2	(0.0, 0.5)	0.5	(0.4, 0.5)***

Coefficients are adjusted for sex and age by multiple linear regression.

95% CIs are shown in parentheses.

*: *P *< 0.05, **: *P *< 0.01, ***:*P *< 0.001.

**Table 4:** Multiple linear regression coefficients of smoking cessation for changes in metabolic syndrome parameters after smoking cessation adjusted by change in body mass index.

	**Men**		**Women**		**Total**	
	**Coefficient**	**95% CI**	**Coefficient**	**95% CI**	**Coefficient**	**95% CI**
***All Subjects***						
(quitter 558, non-quitter 5314)						
						
**HDL cholesterol (mg/dL)**	3.4	(2.7, 4.1)***	1.8	(-0.8, 4.5)	3.2	(2.6, 3.9)***
**Fasting triglyceride (mg/dL)**	-8.2	(-15.5, -0.9)*	4.0	(-9.2, 17.3)	-7.1	(-13.9, -0.3)*
**Fasting serum glucose (mg/dL)**	1.8	(1.2, 2.5)***	2.4	(0.3, 4.4)*	1.9	(1.2, 2.5)***
**Systolic blood pressure (mmHg)**	1.5	(1.0, 2.0)***	-0.5	(-4.1, 3.1)	2.9	(1.8, 4.0)***
**Diastolic blood pressure (mmHg)**	2.1	(1.2, 3.0)***	0.0	(-2.9, 2.8)	1.9	(1.1, 2.7)***
						
***MetS***						
(quitter 33, non-quitter 152)						
						
**HDL cholesterol (mg/dL)**	4.1	(1.5, 6.6)**	39.1	(-774.3, 852.4)	4.2	(1.7, 6.8)**
**Fasting triglyceride (mg/dL)**	-34.7	(-70.6, 1.2)	90.2	(-2383.4, 2563.9)	-36.5	(-72.0, -1.1)*
**Fasting serum glucose (mg/dL)**	2.0	(-1.0, 4.9)	37.7	(-1304.6, 1379.9)	2.1	(-0.8, 5.1)
**Systolic blood pressure (mmHg)**	2.6	(-3.0, 8.2)	-33.6	(-216.6, 149.4)	2.5	(-3.1, 8.0)
**Diastolic blood pressure (mmHg)**	1.8	(-2.4, 5.9)	47.2	(-720.1, 814.4)	2.0	(-2.1, 6.1)
						
***without MetS***						
(quitter 525, non-quitter 5162)						
						
**HDL cholesterol (mg/dL)**	3.3	(2.6, 4.0)***	1.6	(-1.1, 4.3)	3.2	(2.5, 3.9)***
**Fasting triglyceride (mg/dL)**	-7.2	(-14.6, 0.3)	5.2	(-8.2, 18.5)	-6.0	(-13.0, 0.9)
**Fasting serum glucose (mg/dL)**	1.7	(1.0, 2.4)***	2.1	(0.1, 4.2)*	1.7	(1.1, 2.4)***
**Systolic blood pressure (mmHg)**	3.2	(2.0, 4.3)***	-0.5	(-4.1, 3.2)	2.8	(1.7, 4.0)***
**Diastolic blood pressure (mmHg)**	2.1	(1.2, 3.0)***	-0.3	(-3.2, 2.6)	1.8	(1.0, 2.7)***

Coefficients are adjusted for sex, age and change in BMI by multiple linear regression.

95% CIs are shown in parentheses.

*: *P *< 0.05, **: *P *< 0.01, ***:*P *< 0.001.

To clarify the factors contributing to the elevation of MetS risk among smoking quitters, we calculated the distribution of fulfilled MetS criteria components before and after quitting smoking among quitters who got MetS in the second year (n = 33). We found that each MetS criterion turned positive nearly equally after quitting smoking: 15 subjects became hypertensive, 13 subjects became hyperlipidemic (while 2 previously hyperlipidemic subjects became nonhyperlipidemic), and that 13 subjects developed impaired fasting glucose, as for minor criteria (Table 5).

**Table 5:** Components of metabolic syndrome criteria (hypertension: H, hyperlipidemia: L, impaired fasting glucose: G) among 33 examinees (32 males and one female) who became metabolic syndrome after smoking cessation.

**BMI < 25 before cessation (n = 20)**						
Component before cessation			Component after cessation			***n***
**H**	**L**	**G**	**H**	**L**	**G**	
		
-	-	-	+	+	-	2
-	-	-	-	+	+	1
-	-	-	+	+	+	1
+	-	-	+	+	-	2
+	-	-	+	-	+	1
-	+	-	+	+	-	2
-	+	-	+	-	+	1
-	+	-	-	+	+	1
-	-	+	+	-	+	1
-	-	+	-	+	+	2
+	+	-	+	+	-	4
+	+	-	+	-	+	1
+	+	-	+	+	+	1

**BMI ≥ 25 before cessation (n = 13)**						

Component before cessation			Component after cessation			***n***
**H**	**L**	**G**	**H**	**L**	**G**	
		
-	-	-	+	-	+	1
-	-	-	-	+	+	2
-	-	-	+	+	+	1
+	-	-	+	+	-	2
+	-	-	+	-	+	1
-	+	-	+	+	-	4
-	-	+	+	-	+	1
-	+	-	+	+	+	1

Table 6 shows the risk of MetS according to the combined status of smoking cessation and alcohol consumption. When non-quitters who does not drink alcohol were defined as the reference, smoking quitters who drinks alcohol showed statistically significant risk increase in MetS (aHR = 2.42, 95% CI: 1.48–3.94, *P *< 0.001). The interaction of quitting smoking and drinking was 2.03 (95% CI: 0.78–3.94, *P *= 0.146).

**Table 6:** Adjusted hazard ratio (aHR)^a ^and 95% confidence intervals (95% CIs) of metabolic syndrome according to the combined status of smoking cessation and alcohol consumption.

		**Non-drinker**	**Drinker**
**All Subjects**			
**Non-quitters**	***n***	1380	3910
	**MetS**	40	112
			
	**aHR**	1	0.98
	**95% CI**	Reference	0.68–1.41
			
**Quitters**	***n***	174	383
	**MetS**	6	27
			
	**aHR**	1.24	2.42^b^
	**95% CI**	0.52–2.92	1.48–3.94***
			
**Men**			
**Non-quitters**	***n***	1216	3734
	**MetS**	38	107
			
	**aHR**	1	0.94
	**95% CI**	Reference	0.65–1.36
			
**Quitters**	***n***	137	358
	**MetS**	5	27
			
	**aHR**	1.18	2.46^c^
	**95% CI**	0.46–2.99	1.50–4.02***
			
**Women**			
**Non-quitters**	***n***	164	176
	**MetS**	2	5
			
	**aHR**	1	2.59
	**95% CI**	Reference	0.50–13.34
			
**Quitters**	***n***	37	25
	**MetS**	1	0
			
	**aHR**	2.13	0
	**95% CI**	0.19–23.56	-

^a ^Adjusted for sex and age (all subjects), or age (men and women).

^b ^Interaction = 2.03 (P = 0.146).

^c ^Interaction = 2.27 (P = 0.116)

*: *P *< 0.05, **: *P *< 0.01, ***:*P *< 0.001.

We also examined changes in the MetS parameters among drinkers with those among non-drinkers to investigate the factors influenced by drinking by multiple linear regression model, which revealed that none of the parameters showed statistically significant difference in change between the two groups (data not shown).

To evaluate the long-term effect of smoking cessation on the risk of MetS, we checked the aHR of MetS among those who maintained their nonsmoking status for one or two additional years during follow-up. For those who maintained their nonsmoking status for another year (n = 245 among 3,254 examinees), the aHR of MetS was 1.28 (95% CI: 0.69–2.38, *P *= 0.440) (aHR = 1.65, 95% CI: 0.71–3.87, *P *= 0.245 in years 2003–2005 [n = 109 among 1,609 examinees]; aHR = 1.01, 95% CI: 0.41–2.52, *P *= 0.981 in years 2004–2006 [n = 136 among 1,645 examinees]), and for those who maintained their nonsmoking status for two additional years (in years 2003–2006) (n = 28 among 1,496 examinees), the aHR of MetS was 0.74 (95% CI: 0.10–5.37, *P *= 0.769).

## Discussion

Our study result revealed that smoking cessation increased the risk of MetS, and alcohol intake might possibly add to this risk elevation in male smoking quitters.

It has been reported that the mechanisms in which smoking cessation promotes weight gain is explained by increased energy intake, decreased resting metabolic rate, decreased physical activity, and increased lipoprotein lipase activity [9-12]. It is also reported that nicotine exerts its influence on lipid metabolism through the antiestrogenic effects, inducing lypolysis by stimulating the sympathetic nervous system, leading to a consequent increase in plasma free fatty acids [13,14].

In this study, we observed the higher aHR of MetS in smoking quitters than in non-quitters, which seemingly suggested the effect of smoking cessation in the occurrence of MetS. Our further analyses about the MetS parameters showed that levels of HDL-C, FSG, sBP and dBP, and BMI elevated significantly after smoking cessation. To exclude the effect of weight gain and evaluate the true effect of smoking cessation on the risk of MetS, we also calculated the differences in MetS parameters before and after smoking cessation adjusted by BMI increase, which remained significant. In this analysis, significantly lower fasting TG level among smoking quitters than in non-quitters (*P *< 0.05) was observed. This might reflect some early beneficial effect of smoking cessation, in accordance with the conclusions of a review of 54 published studies, which supported a significant dose-response effect between smoking and increased serum TG levels [15]. Although our further analyses among subjects with MetS detected significant difference only in HDL-C levels, FSG levels and BMI, which might be partly attributable to the relatively small number of the subjects, our examination of MetS criteria positive rates before and after quitting smoking revealed that only a part of the subjects getting MetS are explained by weight gain (6 of 33 subjects are attributable to weight gain), and the rest of them are due to acquiring other MetS criteria components. As for the association between smoking cessation and these MetS related factors, following facts are known. The fact that smoking cessation promotes weight gain is already well known [9-11], and our study results confirmed this relation. It is also reported that BP elevation and increased prevalence of hypertension is observed after smoking cessation [16-18]. These BP elevations after smoking cessation are supposed to be explained by increase in weight due to increase in food intake, or increase in stress [16]. Recent study in Korea revealed cigarette smoking as one of the modifiable risk factors for type 2 DM, which demonstrated the transient increase in the risk of diabetes with smoking cessation that remained significant after adjustment for weight change [19]. It is also shown that smoking decreases HDL-C levels through the decrease in lecitin cholesterol acyl-transferase (LCAT) and alteration in the activities of cholesterol ester transfer protein (CETP) and hepatic lipase (HL) [20,21]. Our study results were in the same trends to the previous reports, suggesting that we should take measures not to expose smoking quitters to these adverse effects [22], although these effects seem to be only transient.

To date, some groups examined the association between alcohol intake and risk of MetS [3,4,23,24]. Most of them resulted in insignificant association, or even some protective effect of alcohol against MetS was observed. In this study, we did not observe significantly higher risk of MetS after smoking cessation among drinkers than among non-drinkers (data not shown). However, our combined analysis of smoking cessation and alcohol intake revealed that those who quitted smoking and drank alcohol were at highest risk of MetS, and possible interaction between smoking cessation and alcohol intake was observed although statistically insignificant, suggesting that alcohol intake added to the detrimental effect of smoking cessation on the occurrence of MetS. Our further analyses about the differences in each MetS parameters according to the alcohol consumption status among smoking quitters revealed the tendency that the levels of HDL-C, sBP and dBP were higher in alcohol drinkers, although none of which were statistically significant (data not shown). The seemingly contrast findings of the statistically insignificant differences in the levels of these MetS parameters according to alcohol intake status among smoking quitters or the insignificant interaction between drinking and quitting smoking compared with the significant increase in risk of MetS among quitters who drank alcohol shown in Table 6 might be attributable to the relatively small number of the subjects who quitted smoking and drank alcohol. Meanwhile, whereas recent reports indicate the harmful effect of alcohol intake on MetS, the majority of the reports also support the potential beneficial effect of alcohol intake, especially in mild to moderate amount of alcohol consumption [5,6,23]. This beneficial effect of alcohol intake was not detected in this study; however, there is a possibility that additional analyses according to the amount of alcohol intake might provide such evidence, although they could not be performed because the information of the actual amount of alcohol intake was not available in the present study. Further examination with larger number of subjects or detailed information of alcohol intake is required to clarify the precise effect of alcohol intake on MetS among smoking quitters.

In Table 3 and 4 it is indicated that smoking cessation in the subjects developing MetS is associated with a beneficial effect on HDL-C and TG, which appear to be in contrast with the general result of this study *i.e*. smoking cessation increases the risk of MetS. This discrepancy would be due to the existence of a few quitters developing MetS whose TG levels greatly decreased (TG levels decreased in 13 of 33 quitters developing MetS, among whom TG decreased more than 300 mg/dL in two quitters) compared with the small increase among the rest of the quitters (less than 200 mg/dL increase in 20 of 33 quitters). Those subjects with greater decrease in TG levels would be the early appearance of the beneficial effect of quitting smoking, while the TG levels still increased in nearly two-thirds (20 of 33) of the quitters developing MetS. The beneficial effect of smoking cessation on HDL-C seemed to have been offset by these increases in TG levels.

There are several limitations in this study. First, the imprecise definition of smoking and smoking cessation and the lack of quantification of smoking and alcohol intake are the shortcomings of this study. Other lifestyle factors which might be associated with the risk of MetS like exercise amount or frequency were not available, which also decreased the potential of this study. The lack of information about blood pressure medication at baseline is another important limitation. Further investigations with better study design are expected. The second limitation is concerning the definition of MetS. We adopted the Japanese criteria defined by JASSO [3], because it is popular and widely used in practice in Japan, and is shown to be as predictable of CHD in Japanese as other world's three representative criteria (the National Cholesterol Education Program Adult Treatment Panel III (NCEP-ATP III), American Heart Association in conjunction with the National Heart, Lung, and Blood Institute (AHA/NHLBI), and International diabetes federation (IDF) criteria) [25], and some study demonstrate that Japanese criteria correlate better with CHD in elderly Asian women than these three criteria [26], although we should recognize that this Japanese definition is still one of several premature definitions requiring verification with further prognostic evidence. In the Japanese criteria obesity is defined with WC, but in this study we used BMI instead of WC. In some study, the effects of WC and BMI on cardiovascular risks are similar, but not completely the same; increases in BMI were associated with increased risk of hypertension, dyslipidemia and MetS as well as WC, while risk of diabetes was only associated with WC [27], suggesting that we have to be careful in interpreting the results. Meanwhile, a number of studies have shown that BMI is an effective predictor of type 2 DM or other metabolic disturbances [28], and JASSO recently reported subjects with multiple metabolic risk factors can be detected with the best combination of sensitivity and specificity by a BMI of 25 [29]. Although the 2-hour oral glucose tolerance test (2-h OGTT) is commonly used for accurate diagnosis of impaired fasting glucose, we defined impaired fasting glucose as FSG ≥ 110 mg/dL in this study because the 2-h OGTT was not carried out in most of the study subjects, and it is not part of the NCEP-ATP III, AHA/NHLBI, IDF or Japanese criteria of MetS. A subset of analyses in which BMI is not used as part of the definition of MetS, in which changes in MetS incidence between quitters and non-quitters are adjusted by changes in BMI, might reveal more of the effects of quitting smoking on the risk of MetS independent of BMI change; however, we could not perform these analyses because there were no other effective ways to measure obesity (such as WC) in this dataset. Further investigation of the effects of smoking cessation on the risk of MetS with the definition not including BMI is required. Another limitation is concerning the sample size of this study. The sample size for those who smoked at baseline but quitted smoking at the subsequent checkup, as well as the number of women subjects or the number of subjects used in the analyses of smoking cessation with the risk of MetS over longer follow-up times, is rather small to have a reliable finding, which requires further investigation of this association with a larger number of study subjects in the near future.

Our finding revealed that smoking cessation significantly raised the risk of MetS, and alcohol intake might add to this risk elevation in male smoking quitter. The present study gives us the lesson that medical practitioners should take care of smoking quitters not to gain weight or not to suffer from MetS, by giving them instructions about appropriate amount of exercise, proper energy intake or calorie restriction, along with alcohol cessation.

Our additional analyses of longer follow-up periods showed that smoking cessation had no significant long-term influence on the risk of MetS, suggesting that any detrimental effect of quitting smoking on the risk of MetS only happens over a short period; however, we should be aware of this possibility when promoting smoking cessation. Obviously, the clear beneficial effect of quitting smoking on the prevention of cancer in various organs, cardiovascular diseases in addition to MetS [3] is more important than avoiding this transitory potential for MetS.

## Conclusion

This study revealed that smoking cessation transiently increased the risk of MetS, and alcohol intake might add to it. Further investigation with better study design or in other study groups is required to confirm our findings.

## Competing interests

The authors declare that they have no competing interests.

## Authors' contributions

A H performed the statistical analysis and prepared the manuscript. A K, A T and S K provided the data and reviewed the manuscript. N H was responsible for the study design and coordination, and reviewed the manuscript critically. All authors read and approved the final manuscript.

## Pre-publication history

The pre-publication history for this paper can be accessed here:


